# Machine and deep learning based on magnetic resonance imaging to segment glioblastoma and predict the spread of recurrence: a multicenter retrospective protocol

**DOI:** 10.3389/fneur.2026.1818594

**Published:** 2026-07-10

**Authors:** Luana Conte, Erica Lo Turco, Rosaria V. Abbritti, Caterina Accettura, Giuseppe Raso, Edvige Iaboni, Ugo De Giorgi, Giorgio De Nunzio, Donato Cascio, Maria Caffo

**Affiliations:** 1 Department of Physics and Chemistry “E. Segrè”, University of Palermo, Palermo, Italy; 2 Laboratori Nazionali del Sud, National Institute for Nuclear Physics (INFN), Catania, Italy; 3 Laboratory of Interdisciplinary Research Applied to Medicine (DReAM), University of Salento & ASL Lecce, Lecce, Italy; 4 Azienda Ospedaliero Universitaria “R. Dulbecco”, Catanzaro, Italy; 5 Service de Neurochirurgie Hôpital “Lariboisière”, Paris, France; 6 Department of Experimental Medicine, University of Salento, Lecce, Italy; 7 Unit of Neurosurgery, Department of Biomedical and Dental Sciences and Morphofunctional Imaging, University of Messina, Messina, Italy; 8 Department of Mathematics and Physics “E. De Giorgi”, University of Salento, Lecce, Italy; 9 National Institute for Nuclear Physics (INFN), Lecce, Italy

**Keywords:** deep learning, glioblastoma, machine learning, radiomics, recurrence

## Abstract

**Background:**

Glioblastoma (GB) remains one of the most aggressive brain tumors, with limited survival and high recurrence rates. In most cases, GB recurrence occurs locally, either on the residual tumor after surgery or within 2 cm of the resection cavity—but in rarer cases, tumor cells can spread beyond this margin, leading to distant recurrence. By *spread*, we refer to the spatial dissemination of tumor cells beyond the typical local site, which can involve distant brain regions or even the leptomeninges, significantly impacting treatment planning and surgical decision-making. This study proposes the application of Machine Learning (ML) and Deep Learning (DL) approaches to MRI data from GB patients in the preoperative phases, aiming to develop predictive models to predict the extent of recurrence spread, on the integrated analysis of clinical, imaging, and instrumental data. Additionally, we plan to design a (semi) automatic segmentation tool for tumor delineation in MRI, which will support both the implementation of the study and serve as a standalone instrument to standardize volume measurement in neuroimaging.

**Methods and analytics:**

A multicenter retrospective collection of clinical and radiological variables will be performed for all eligible GB patients. Variables will include demographic, surgical, pathological, and preoperative MRI features. Predictive modelling will use classical ML algorithms (e.g., Random Forest, SVM, Multilayer perceptron, etc.) and a 3D U-Net architecture for DL-based image segmentation. Dimensionality reduction (PCA, LASSO, etc.) will be used to prevent overfitting and improve model generalizability. Model performance will be assessed through Area Under the Curve (AUC), P-R curve, F-score, accuracy, sensitivity, specificity, confusion matrix, and Dice score for segmentation.

**Discussion:**

The development of Artificial Intelligence (AI)-based predictive models for GB is expected to provide a major contribution to outcome prediction, early targeted interventions, and personalized care. These tools may support optimized resource allocation, reduce healthcare costs, and improve patient and family outcomes. The findings from this study will serve as a foundation for a future prospective multicenter validation study.

## Introduction

Glioblastoma (GB) is a highly infiltrative and aggressive neoplasm with a very poor prognosis and a high propensity of recurrence (1, 2). Despite multidisciplinary therapy approaches that include maximal safe resection followed by radiotherapy and chemotherapy, a long-lasting local tumor control cannot be achieved in most GB patients (3, 4).

A key challenge lies not only in detecting recurrence but in anticipating its spatial distribution, with an eventual distant spread. The location of recurrence is generally unpredictable and influenced by multiple factors, including surgical resection margins, as well as biological heterogeneity of the GB. In most cases, local recurrence within 2 cm of the resection cavity is inevitable. Brain tumors, including GB, rarely recur at anatomically distant sites; rather, they tend to exhibit a progressive spatial spread from the original tumor bed, extending beyond the immediate surgical margins. By *spread*, we refer to the gradual infiltration of tumor cells into adjacent or more distant brain regions, beyond the typical local recurrence zone. Therefore, it is the pattern of tumor cell dissemination, rather than the occurrence of isolated distant relapse, that would be most valuable to predict, as it ultimately determines the site and extent of recurrence in relation to the previous surgical cavity.

The choice between subtotal and gross total resection should be carefully tailored based on the lesion’s location, particularly whether it involves eloquent brain areas such as motor pathways, language areas, or other critical structures, in order to minimize the risk of postoperative or delayed neurological deficits. Additionally, the surgical approach is primarily guided by meticulous preoperative planning, which includes accurate anatomical localization of the tumor. Intraoperative neuromonitoring further informs surgical decisions, along with the prediction of the recurrence’s site, which can influence both the extent and trajectory of resection. Given the prognostic and therapeutic implications, particular attention should be paid to the precise anatomical localization of the predicted relapse. Predicting the site of recurrence — particularly whether it will occur locally, at the site of residual tumor or distally — is crucial for surgical planning as well as for successive oncological treatments. Knowing in advance where a recurrence is likely to occur allows the neurosurgeon to adopt a more extensive and aggressive resection strategy during the initial surgery, thereby improving the chances of local disease control (5–7). This consideration becomes especially relevant in cases where recurrence tends to arise within the same anatomical compartment or along resection margins. Moreover, predicting and precisely localizing tumor recurrence can significantly impact radiotherapy planning. If recurrence is expected beyond the tumor bed—the usual target of adjuvant irradiation—expanding radiation fields or adjusting treatment volumes may be warranted. Emerging evidence supports that personalized radiotherapy strategies can improve therapeutic outcomes.

In this context, there is a growing need for tools capable of forecasting the direction and extent of tumor spread. Recent advances in Artificial Intelligence (AI), particularly traditional Machine Learning (ML) and Deep Learning (DL), have demonstrated potential in supporting medical data analysis (2). These approaches aim to develop predictive models that integrate clinical, surgical, and imaging data to anticipate specific outcomes, guide therapeutic decisions, and improve patient management (8, 9).

In recent years, ML-based methods have been increasingly applied to the study of GB (2). However, to the best of our knowledge, they have not yet been successfully employed to predict the spatial pattern of GB spread, which would represent a critical step toward pre-emptive and personalized therapeutic planning.

Moreover, accurate and reproducible delineation of tumor volumes on MRI is a critical prerequisite for both model training and clinical decision-making. Therefore, alongside the development of predicting models, we will design and validate a semi-automatic segmentation tool based on a 3D U-Net architecture, to standardize tumor volume measurement and support downstream AI analyses.

## Methods and analysis

### Study design

This retrospective study will be conducted at different Italian Units of Neurosurgery for clinical data and images acquisition, with all ML and DL analyses performed at the Department of Physics and Chemistry of the Università of Palermo (Italy) and the Department of Mathematics and Physics of the University of Salento (Italy).

**Figure 1:**
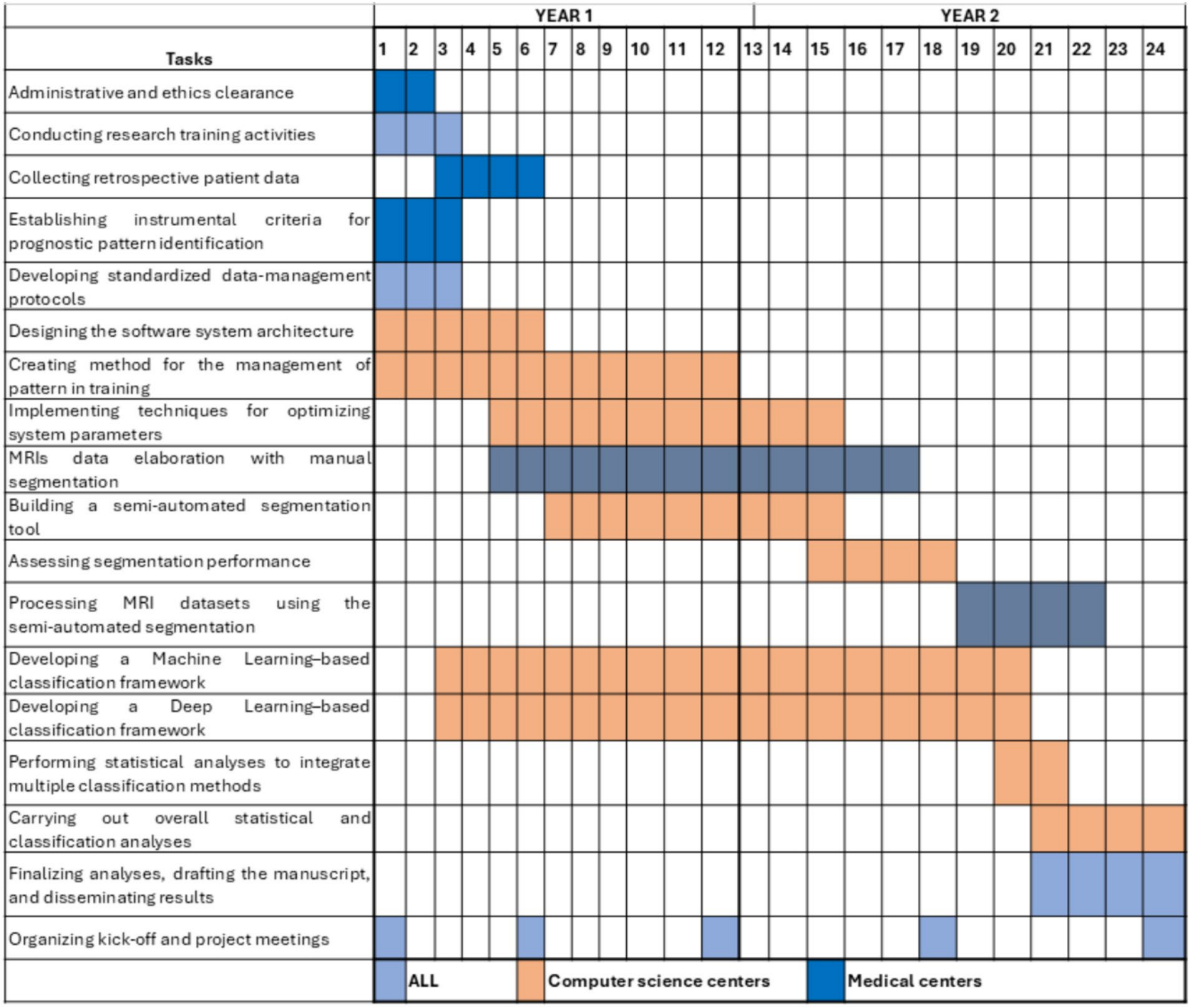
Gantt chart outlining the 24-month project timeline and task allocation. Key activities include: administrative and ethics clearance; research training; retrospective data collection; definition of prognostic parameters and data-management protocols; software system design; development and validation of manual and semi-automated MRI segmentation tools; implementation of Machine Learning and Deep Learning classification frameworks; statistical integration of methods; overall analysis, manuscript drafting, and result dissemination; and recurring project meetings. Colour coding denotes responsibility: grey bars indicate tasks conducted by all partners, orange bars those led by the computer science centres, and blue bars those led by the medical centres.

The project unfolds in two successive phases spanning a total of 24 months (Figure 1). During the first 6 months, we will secure all necessary administrative approvals and ethics committee clearances, complete team training, and assemble the retrospective clinical and imaging dataset. At the same time, we will define the key instrumental parameters for prognostic feature extraction, establish standardized protocols for data handling and quality control, and design software architecture to support subsequent ML and DL workflows.

From month 7 through month 24, the focus shifts to development, integration and validation of the prototype ML- and DL-based predictive prognostic models. Retrospective data will be used to train separate ML and DL models aimed at predicting the spread of GB recurrence. These models will be refined iteratively and integrated into a unified framework of prototype ML- and DL-based predictive prognostic models, which will then be applied to prospective data as it becomes available.

Concurrently, an automated segmentation software prototype will be deployed and optimized throughout this period. All software components, from preprocessing pipelines to the predictive prognostic models and segmentation modules, will undergo continuous testing and improvement.

### Study population and sample size

We will retrospectively identify all consecutive patients with histologically confirmed aggressive high grade glioma who experienced a recurrence and underwent treatment between January 2014 and December 2020.

This time frame was selected to ensure an adequate follow-up duration for most patients, allowing for meaningful analysis of recurrence patterns and survival outcomes. Based on institutional records, approximately 200 patients are expected to meet the initial screening criteria.

**Figure 2:**
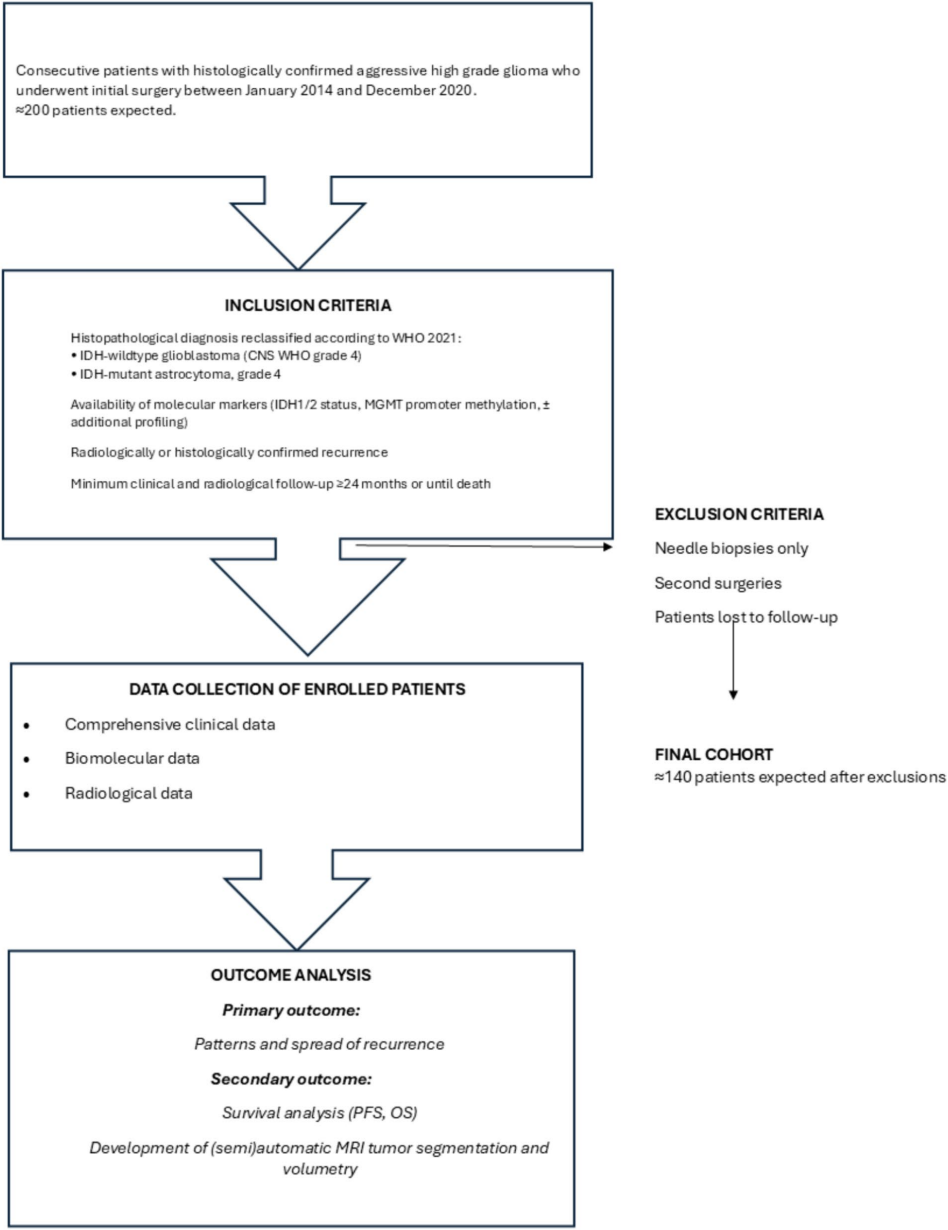
Study flowchart showing patient selection, inclusion/exclusion criteria, and outcome analysis. IDH, isocitrate dehydrogenase; MGMT, O6-methylguanine-DNA methyltransferase; PFS, progression-free survival; OS, overall survival.

Enrollment will be based on the following inclusion criteria (Figure 2):

- Histopathological diagnosis of glioblastoma or diffuse astrocytoma, reclassified according to the 2021 WHO CNS tumor classification:

i IDH-wildtype glioblastoma (CNS WHO grade 4), or
ii IDH-mutant astrocytoma, grade 4.

- Availability of molecular markers (IDH mutation status, MGMT promoter methylation, etc.), when tested.
- Radiologically or histologically confirmed recurrence.
- Sufficient clinical and radiological follow-up, defined as a minimum of 24 months or until documented disease progression or death.

By including cases up to December 2020, we ensure a follow-up window extending at least to 2024–2025, which is crucial given the potential for long survival in patients with IDH-mutant tumors.

Needle biopsies, second surgeries, and patients lost to follow-up will be excluded. Calculating an approximate 30% dropout rate due to the exclusion criteria, we would enroll around 140 patients in the final analysis.

While traditional power calculations and formal sample size justifications are challenging for exploratory high-dimensional radiomic studies, a final cohort of approximately 140 patients is considered methodologically adequate for classical Machine Learning applications. To mitigate the risk of overfitting and directly address the potential class imbalance between local and distant recurrences, we will implement data augmentation and synthetic resampling techniques (such as the Synthetic Minority Over-sampling Technique, SMOTE) on the extracted radiomic features, ensuring robust and generalizable model training.

### Data collection

For each enrolled patient, we will extract clinical and imaging parameters from electronic medical records. Demographic and clinical variables will include age, tumor localization and laterality, presence of residual tumor after surgery, type of adjuvant treatment, type of recurrence (local or residual, distant, or combination), delay of relapse and biomolecular markers of the tumor.

### Radiological parameters

Imaging will be performed using Synapse PACS and Synapse 3D (FUJIFILM Medical Systems USA, Inc.). Preoperative MRIs of all patients will be collected using contrast-enhanced T1-weighted sequences. Radiological assessment of recurrence will be performed according to the RANO (Response Assessment in Neuro-Oncology) criteria, which consider both radiological and clinical parameters:

- Complete Response (CR): disappearance of all enhancing disease for at least 4 weeks, no new lesions, and stable or improved clinical status, off corticosteroids.
- Partial Response (PR): ≥50% decrease in the size of measurable enhancing lesions compared to baseline, sustained for at least 4 weeks, with no new lesions, stable/improved clinical condition, and stable or reduced corticosteroid use.
- Stable Disease (SD): radiological findings that do not meet the criteria for CR, PR, or PD, i.e., no significant tumor shrinkage or progression, with stable clinical status.
- Progressive Disease (PD): ≥25% increase in the size of enhancing lesions, appearance of new lesions, or clear clinical deterioration not attributable to other causes.

A local recurrence will be defined as tumor regrowth within 2 cm of the resection cavity. Residual recurrence will be defined as an increase in the residual neoplasm, while residual tumor will be defined as persistence of contrast-enhancing tumor tissue after surgery. Distant recurrence will be defined as new tumor lesions occurring >2 cm from the resection cavity. For the purpose of the binary classification framework, mixed or multifocal recurrences presenting both local progression and distant new lesions will be assimilated into the distant recurrence class.

To mitigate the inherent variability of a multicenter retrospective design, all preoperative MRIs will undergo a rigorous standardization and preprocessing pipeline prior to tumor segmentation and feature extraction. Initially, images will be subjected to N4 bias field correction to resolve magnetic field inhomogeneities. Subsequently, to ensure spatial uniformity across different scanner vendors and acquisition protocols, all scans will be co-registered to a standard anatomical space and resampled to an isotropic voxel resolution of 1x1x1 mm using appropriate interpolation techniques. Image intensity distributions will also be standardized using Z-score intensity normalization or histogram matching. Finally, to further account for residual scanner-related batch effects on the quantitative data, an advanced statistical harmonization technique, such as the ComBat method, will be applied to the extracted radiomic feature matrices prior to their integration into the machine learning classification framework.

**Figure 3:**
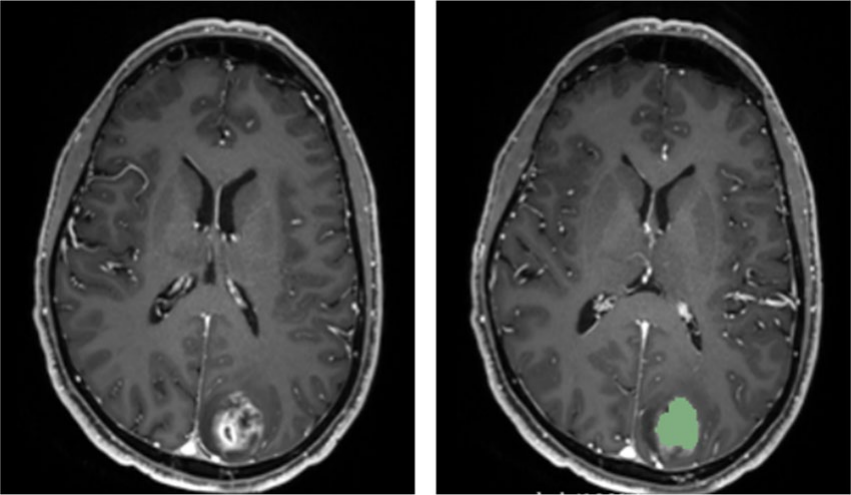
Example axial post-contrast T1-weighted MRI slices of a glioblastoma patient. Left: original image with no annotation. Right: same slice with manual segmentation overlay (green) highlighting the enhancing tumor volume of interest (VOI). This illustrates the region used for subsequent feature extraction and model training.

For the development of the predictive algorithm and the automatic segmentation task, MRIs will be processed using MITK-MI brain software with the NIfTI data format. Tumor regions will be manually delineated on each slice using freehand Volume of Interest (VOI), and the software will calculate total lesion volume by summing the area of all Regions of Interest (ROIs), multiplied by slice thickness and interslice gap (Figure 3).

### Outcome

#### Primary outcome

Prototype ML- and DL-based predictive prognostic models capable of forecasting the *spread of recurrence*, intended as the predicted site of recurrence relative to the original surgical cavity. In this context, *spread* refers to the spatial distribution of tumor cell regrowth—whether it occurs locally (within 2 cm or on residual tissue) or extends toward more distant brain regions. The first class is Local/Residual recurrence, defined as tumor regrowth strictly within 2 cm of the resection cavity or progressing from residual tumor tissue. The second class is Distant spread, defined as the emergence of tumor cells extending toward more distant brain regions beyond the 2 cm margin, including mixed patterns where both local and distant recurrences are simultaneously present.

#### Secondary outcome

Development of a software system for (semi) automatic segmentation and volume measurement in MRIs.

### Analysis

Data analysis will be performed using ML and DL techniques, both of which fall under the broader field of AI and supervised pattern recognition (10, 11).

ML is a well-established approach that involves algorithms capable of processing and learning from data without being explicitly programmed. Once trained, these algorithms can apply the learned patterns to make data-driven predictions. A typical ML workflow includes several sequential stages: data acquisition and preprocessing, computation of a large number of variables (also referred to as features or attributes, which may be either domain-specific or domain-agnostic), dimensionality reduction, model training and validation, and eventual deployment (12). During training, the ML model identifies patterns and correlations between features and the outcome variable of interest, aiming to generalize this knowledge and accurately predict outcomes even in previously unseen cases.

DL represents a more recent advancement within ML, offering a key advantage in its ability to automatically learn complex, high-level features directly from the data. Unlike traditional ML approaches, which require manual feature engineering by domain experts or extensive trial-and-error processes, DL models progressively extract informative representations in an automated and hierarchical manner. This significantly reduces the dependency on prior domain knowledge and manual intervention in feature selection.

The goal of the ML and DL classification approaches will be to predict the spread of GB recurrence, based on preoperative clinical, radiological, and instrumental data. These models aim to classify patients according to expected recurrence patterns, such as local versus distant regrowth, and provide spatial forecasts that can support surgical planning and treatment personalization.

Data analysis will be conducted using Python and Matlab environments. The computational framework will rely on the integrated and multivariate analysis of features derived from both clinical and imaging data collected during the pre-operative periods.

For MRI image analysis, feature extraction will be performed using the open-source PyRadiomics library, in strict compliance with the Image Biomarker Standardisation Initiative (IBSI) guidelines. To ensure standardization, all images will be resampled to an isotropic voxel size of 1x1x1 mm using B-spline interpolation prior to extraction. Image discretization will be performed using a fixed bin width approach to maintain the absolute intensity meaning of the MRI sequences. The extracted agnostic radiomic features will encompass multiple categories, including 3D shape, first-order statistics, and higher-order texture matrices such as the Gray Level Co-occurrence Matrix (GLCM), Gray Level Run Length Matrix (GLRLM), Gray Level Size Zone Matrix (GLSZM), Gray Level Dependence Matrix (GLDM), and Neighborhood Gray Tone Difference Matrix (NGTDM). To guarantee methodological robustness and address segmentation uncertainty, a reproducibility analysis will be conducted. A representative subset of cases will be independently segmented by two different expert radiologists. The Intra-class Correlation Coefficient (ICC) will be calculated for each extracted feature, and only robust features demonstrating an ICC greater than 0.75 will be retained for the dimensionality reduction and ML modeling phases. To strictly prevent any circular dependency within the study design, the radiomic feature extraction for the predictive classification models will rely exclusively on the manual segmentations performed by the expert neuroradiologists, which serve as the definitive ground truth. The development of the 3D U-Net is designated as a parallel and independent task. The automated segmentation tool will be trained on these manual annotations but will not be utilized to generate the input features for the retrospective predictive models, positioning it solely as a standalone secondary outcome for future prospective applications.

The issue of class imbalance, which may be present in the dataset, will be addressed by incorporating appropriate strategies during model training. Depending on the observed distribution, techniques such as class weighting or resampling (e.g., oversampling the minority class) may be applied to reduce potential bias and improve the model’s ability to detect less represented cases.

After the contouring and segmentation steps, a set of feature descriptors will be extracted from the segmented volumes of interest to enable supervised pattern recognition using ML techniques. Initially, expert radiologists will manually perform the segmentation process. However, as the study progresses, we will also develop a semiautomatic segmentation tool designed to support and streamline the radiologists’ workflow.

In the ML-based analysis, the extracted features will include a combination of specific clinical variables (as detailed in the “Data Collection” and “Radiological Parameters” sections), semantic descriptors derived from diagnostic imaging, and agnostic radiomic features that are independent of anatomical context (13).

Once the clinical and imaging feature vectors are defined, the resulting dataset will contain a large number of variables relative to the number of cases available. This imbalance may lead to what is commonly referred to as the “curse of dimensionality,” a situation in which the high dimensionality of the data reduces classification performance. This occurs because the number of samples required to accurately estimate complex relationships grows exponentially with the number of features.

To mitigate this issue, a dimensionality reduction step will be incorporated. This step will eliminate redundant or irrelevant features and retain only the most informative variables, thereby enhancing both model performance and computational efficiency (14). We will employ feature extraction techniques such as Principal Component Analysis (PCA), Independent Component Analysis (ICA), and Fisher Linear Discriminant Analysis (FLDA), which generate new, reduced-dimensional feature sets from the original data. In parallel, we will apply feature selection methods aimed at identifying a subset of the most predictive features while discarding those that do not contribute meaningfully to classification accuracy.

An added benefit of feature selection lies in improving the interpretability of ML models. By isolating the most relevant features, the process supports human-understandable insights into the decision-making process and helps reduce the so-called “black box” nature often associated with ML algorithms, thus contributing to model explainability (15).

Classification of the extracted feature vectors will be carried out using conventional feed-forward Artificial Neural Networks, Support Vector Machines (SVM), Decision trees, Multi layer perceptron (MLP) (10, 11, 16–18) and other established classification algorithms (19). To enhance predictive performance, ensemble learning methods such as Random Forests will also be explored.

**Figure 4:**
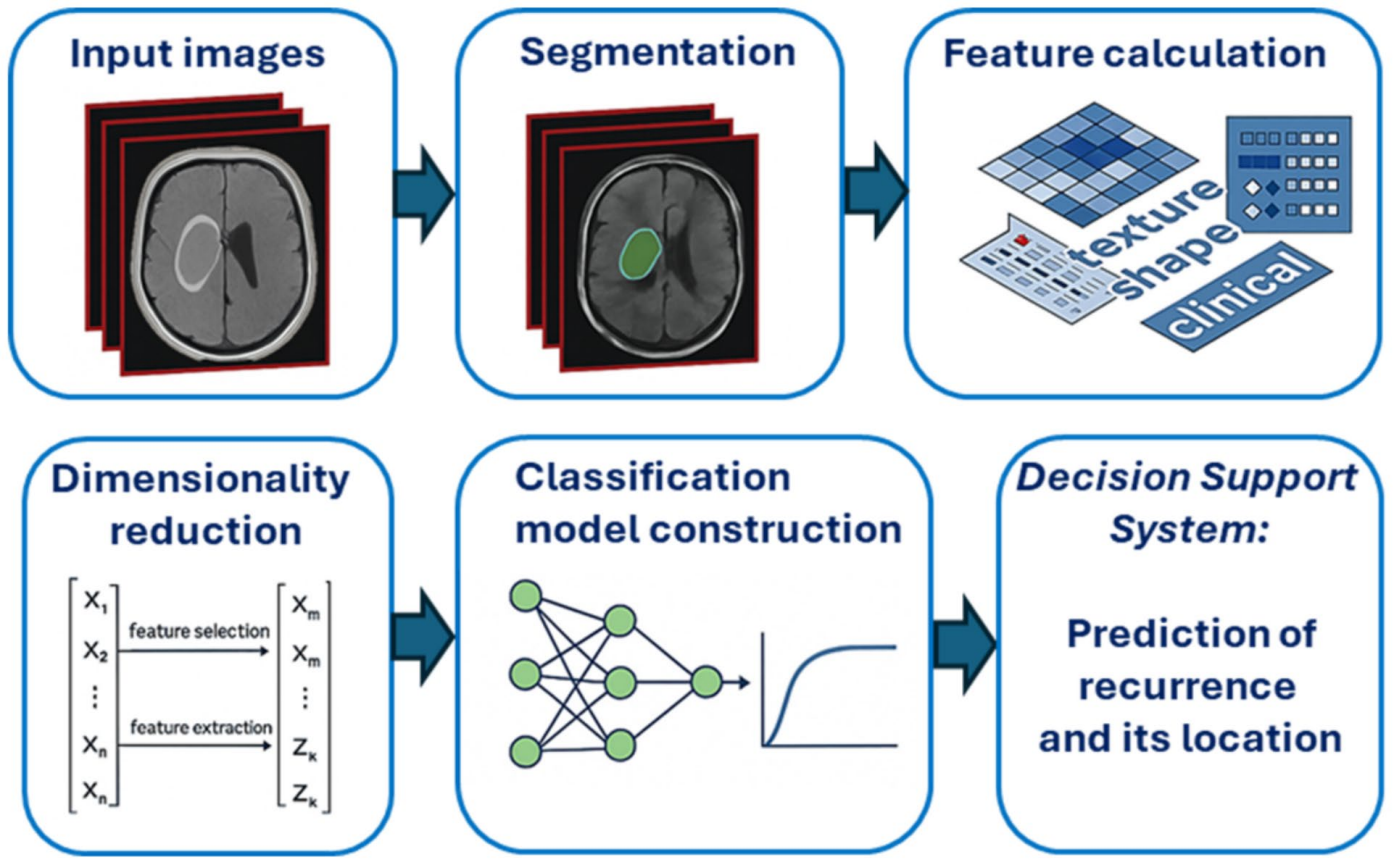
Machine learning pipeline. Workflow diagram of the proposed Radiomics pipeline for Glioblastoma recurrence and location prediction. Preoperative MRI scans are first processed as input images, then undergo segmentation to delineate tumor volumes. Next, quantitative feature calculation extracts agnostic (texture, shape) and semantic (clinical) descriptors. The resulting high-dimensional feature set is trimmed via dimensionality reduction (feature selection and extraction), and a Classification model construction step trains a predictive algorithm. Finally, the Decision Support System: prediction of recurrence and its location integrates the model output to inform clinical follow-up and treatment planning.

To ensure a robust evaluation and mitigate the risk of optimistic performance estimates, model validation will be conducted using a nested repeated stratified k-fold cross-validation framework. This rigorous approach prevents data leakage by explicitly separating hyperparameter optimization, which is executed using grid search or randomized search within the inner cross-validation loop, from the final unbiased performance assessment conducted on the hold-out folds in the outer loop. Crucially, to preserve the strict separation between training and testing cohorts, all data transformation procedures, including dimensionality reduction, feature selection, and the application of data augmentation techniques such as SMOTE, will be fitted exclusively on the training folds and subsequently applied to the corresponding independent validation folds. Model performance will be assessed using Receiver Operating Characteristic (ROC) curves, along with sensitivity and specificity metrics (Figure 4).

**Figure 5:**
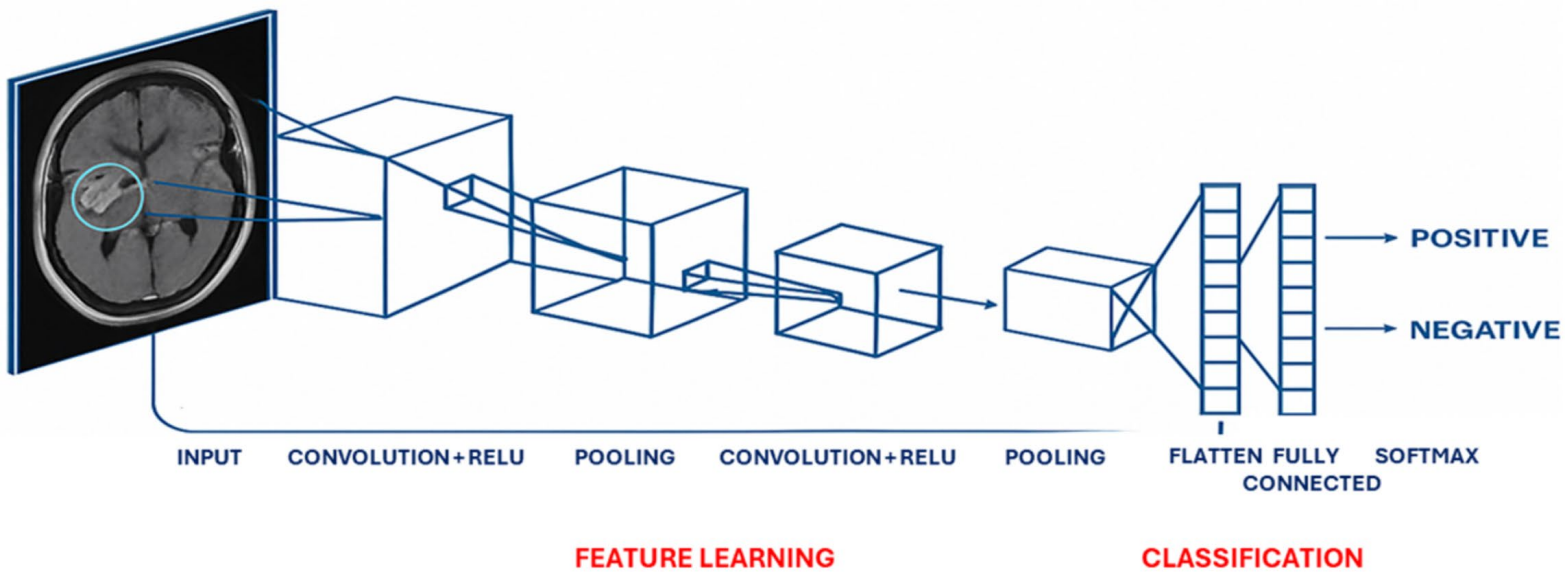
Schematic of the architecture of a convolutional neural network (CNN). A convolutional neural network (CNN) consists of a series of specialized layers that progressively transform and refine the input image into a final classification decision. First, convolutional layers apply learnable filters to extract localized features, and the ReLU (rectified linear unit) activation function is used after each convolution to introduce non-linearity and accelerate training. Pooling layers then downsample the feature maps, reducing spatial dimensions and improving invariance to small shifts. After repeating convolution + ReLU and pooling blocks to build a rich, hierarchical representation, the resulting feature maps are flattened into a single one-dimensional vector. This vector feeds into one or more fully connected layers, where each neuron computes a weighted sum of its inputs to integrate all learned features. Finally, the last fully connected layer uses a Softmax activation to convert raw scores into normalized probabilities, allowing the network to assign the input image to one of the target classes.

Given the sample size boundaries, classical Machine Learning algorithms will serve as the primary and most clinically interpretable analytical framework for the prediction of recurrence spread. The classification capabilities of DL, specifically pre-trained convolutional neural networks (CNNs) such as AlexNet, SqueezeNet, ResNet, and GoogLeNet (Figure 5) (20), will be assessed as an exploratory secondary analysis to evaluate potential performance gains over classical ML These networks will be fine-tuned using different training strategies, including three configurations involving partial weight freezing, as well as training from scratch. Additionally, the CNNs will be used as feature extractors, whereby the learned feature representations are passed to classical ML classifiers, for example SVMs. This hybrid approach leverages the representational power of CNNs while benefiting from the simplicity and efficiency of some ML algorithms, which are particularly suitable for high-dimensional classification tasks such as those involved in this study.

For the segmentation task, we will implement a 3D U-Net architecture (21), which has demonstrated excellent performance in volumetric medical image segmentation. This architecture extends the previously introduced 2D U-Net and is now widely adopted as a state-of-the-art method in the field (22, 23). Fully convolutional neural networks (CNNs) like U-Net have become the dominant strategy for automatic segmentation, particularly due to their ability to perform well even when trained on a limited number of annotated images, a common scenario in medical imaging.

The 3D U-Net architecture is composed of two main components: a contracting path and an expanding path. The contracting path captures high-resolution local features, while the expanding path reconstructs spatial resolution by upsampling global low-resolution features. By concatenating these two types of information at each stage, the network is able to integrate both local detail and broader contextual information. In our case, the 3D U-Net architecture will be specifically trained and optimized for segmenting tumor volumes using contrast-enhanced T1-weighted MRI sequences as the primary input channel. The ground truth annotations will be generated manually by two independent expert neuroradiologists, and baseline inter-rater agreement will be evaluated using the Dice Similarity Coefficient to ensure high-quality training data. To optimize the network weights, we will employ the Adam optimizer coupled with a dynamically scheduled learning rate. The training process will be guided by a hybrid loss function combining Dice Loss and Cross-Entropy Loss to effectively handle the volumetric class imbalance between the enhancing tumor regions and the background brain tissue. Furthermore, to prevent overfitting and effectively leverage the massive amount of voxel-level data contained within the 140 whole-brain 3D MRI volumes, the network will be trained utilizing extensive spatial data augmentation techniques, such as random rotations, scaling, and elastic deformations. These combined structural parameters and augmentation strategies are explicitly designed to maximize the model’s generalization capabilities across different scanning protocols.

## Discussion

In the era of precision medicine, integrating diverse types of data from multiple sources is essential for delivering comprehensive patient care (24). Given the intrinsic complexity and heterogeneity of GB, this pathology is particularly suitable for an integrated analysis using ML- and DL-based approaches applied to a wide array of clinical, instrumental, and imaging data.

Over time, medical imaging has gained a central role in monitoring treatment response, assessing toxicity, and more recently, in predicting outcomes that can guide therapeutic strategies (25–32). The application of AI methods is expected to provide a significant advancement in the clinical management of GB patients.

Despite this evolution, imaging interpretation still carries a degree of subjectivity and inter-operator variability (33). Segmentation, in particular, remains a fundamental yet manual process for assessing tumor volume and shape. Most commonly used medical imaging platforms do not provide dedicated segmentation capabilities, which forces clinicians to rely on manual contouring. This is time-consuming, operator-dependent, and prone to error, making the results difficult to compare across observers.

The development of an automatic or semiautomatic segmentation tool specifically designed for GB lesion analysis in MRIs would represent a relevant innovation in clinical practice. Such a system would enable standardized tumor volume assessment and contribute to a more accurate and efficient diagnostic process. While the field of neuro-oncology has already significantly benefited from highly advanced automated glioblastoma segmentation frameworks, such as those associated with the BraTS challenges and the state-of-the-art nnU-Net architecture, the deployment of our customized software will specifically streamline our retrospective and prospective multicenter data collection. By integrating seamlessly into our specific clinical and analytical workflow, this tailored tool will support the creation of robust predictive models and ultimately enhance patient management. The success of this study is strongly linked to its multidisciplinary design, which involves collaboration between clinical neuro-oncologists and statisticians, physicists, computer scientists. The convergence of these diverse expertise areas ensures a comprehensive and innovative approach to the research question.

This retrospective investigation could represent the foundation for a new line of research focused on the prediction of GB recurrence presence and localization. In the future, we aim to validate our findings through a prospective, multicenter cohort study involving a larger population.

While ML and DL both fall under the broad umbrella of AI, ML refers to classical techniques that learn from data to make informed decisions. The limitations of ML often stem from the quality and completeness of training data. DL approaches attempt to overcome these limitations by leveraging pre-trained models that transfer learned features from other domains and fine-tune them on smaller, domain-specific datasets.

Although GB is a compelling candidate for ML- and DL-based investigation, the retrospective nature of the study and the rarity of certain recurrence patterns may limit generalizability. Additionally, the intrinsic variability of the disease might not be fully captured due to sample size and potential gaps in data completeness. Nevertheless, the use of electronic medical records and centralized imaging databases is expected to mitigate some of these limitations by providing a more structured and consistent dataset.

Another major challenge involves the high dimensionality of the data. When the number of variables exceeds the number of patients, classification accuracy may suffer due to the “curse of dimensionality,” in which the number of samples needed to achieve a reliable model grows exponentially with the number of features (34). This issue will be addressed using the dimensionality reduction methods described in the “Analysis” section (14).

In recent years, DL models, and in particular convolutional neural networks (CNNs), have gained substantial attention in medical imaging research (35). Their popularity is due not only to their strong classification performance but also to their ability to streamline the entire analytical process by integrating feature extraction within the model training pipeline.

Despite limitations, our cohort is expected to be heterogeneous and to include patients across the full spectrum of disease severity and treatment complexity. This diversity will constitute a strength of the study by improving the generalizability and clinical relevance of the developed models.

## Conclusion

AI-based predictive algorithms are expected to make a substantial contribution to accurate outcome prediction, early targeted interventions, and personalized management of patients with GB. In particular, the development of ML- and DL-based models to identify prognostic patterns, such as the spread of recurrence, represents an exploratory step toward understanding glioblastoma spatial behavior. While these computational tools hold promise, their actual clinical utility, their impact on patient outcomes, and their potential for guiding targeted interventions remain to be rigorously evaluated in future prospective, clinically validated trials. Our experience suggests the potential for ML in identifying recurrence patterns in the data for therapeutic improvement and optimize care pathways. This work represents a further step toward the implementation of precision medicine in the management of GB.
